# Advantages of Ambulatory Blood Pressure Monitoring in Assessing the Efficacy of Antihypertensive Therapy

**DOI:** 10.1007/s40119-015-0043-1

**Published:** 2015-06-16

**Authors:** Alejandro De la Sierra

**Affiliations:** Hospital Mútua Terrassa, Universidad de Barcelona, Terrassa, Barcelona Spain

**Keywords:** Ambulatory blood pressure monitoring, Antihypertensive agents, Blood pressure variability, Circadian blood pressure profile, Hypertension, Morning blood pressure surge, Morning hypertension, Nocturnal hypertension

## Abstract

The cumulative evidence in the past three decades situates ambulatory blood pressure monitoring (ABPM) as a central element in diagnosing and predicting the prognosis of subjects with hypertension. However, for various reasons, this diagnostic and prognostic importance has not been translated in equal measure into making decisions or guiding antihypertensive treatment. Mean 24-h, daytime, and night-time blood pressure estimates, the occurrence of divergent phenotypes between clinic measurements, and ABPM, as well as the main elements that determine blood pressure variability over 24 h, especially night-time dipping, are all elements that in addition to providing evidence for patient prognosis, can be used to guide antihypertensive treatment follow-up enabling greater precision in defining the effect of the drugs. In recent years, specific indices have been developed using 24-h monitoring, evaluate the duration of treatment action, the homogeneity of the effect over the monitoring period, and its possible effects on variability. In future controlled clinical trials on antihypertensive therapies it is necessary to evaluate the effects of those treatments on hard endpoints based on therapy guided by ABPM.

**Electronic supplementary material:**

The online version of this article (doi:10.1007/s40119-015-0043-1) contains supplementary material, which is available to authorized users.

## Introduction

Non-invasive clinic blood pressure (BP) measurement dates back to the early twentieth century and corresponds to the invention of the mercury sphygmomanometer by the Italian physicist Scipione Riva-Rocci, and the description of arterial sounds by the Russian physician Nikolai Korotkoff. Since then, changes in the procedure for clinic measurement have been few and have mostly been based on the appearance of automatic or semi-automatic devices that have used a plethysmographic method instead of the classical auscultatory method described by Korotkoff. Part of this change has been motivated by restrictions on using mercury in health care devices.

The two ways of measuring out-of-clinic BP, self-measurement at home and ambulatory BP monitoring (ABPM), were developed to obtain BP measurements outside health care settings, since those settings have a significant influence on BP in some individuals. Furthermore, especially in the case of ABPM, obtaining a higher number of measurements in a period containing the main sources of BP variability (activity/rest) provides a more precise approximation of an individual’s true BP.

ABPM research has been particularly focused on epidemiological and diagnostic aspects. In this regard, the main indicators obtained during the course of ABPM correlate better with an individual’s organ damage and cardiovascular prognosis [1–4]. Moreover, regarding diagnostic aspects, ABPM has enabled two new phenotypes to be defined, white-coat hypertension (HTN) and masked HTN, both of great clinical interest [5, 6].

Applying ABPM to therapeutic assessment has had less of an impact, both on the part of the investigators as well as on the part of the investment made by the sponsors of antihypertensive drugs and devices. A large part of this is motivated by the fact that the regulatory authorities continue to consider clinic BP measurement as the central element for approving antihypertensive drugs and devices. Nevertheless, the BP measurement indicators obtained using ABPM, the patient phenotypes during hypertensive treatment, dipping patterns, and BP variability over 24 h of monitoring, are of equally high interest when analyzing the effects of antihypertensive therapies [7]. Furthermore, using ABPM enables a single assessment of some aspects of that treatment, such as the duration of action by calculating specific indices that evaluate the duration and the homogeneity of the effect.

This article describes the main indicators, direct or derived, from monitoring itself or combined between ABPM and clinic measurement that may be of interest in patients during antihypertensive treatment. This article is based on previously conducted studies and does not involve any new studies of human or animal subjects performed by the author.

## Mean 24-h, Daytime, and Night-Time BP Estimators

The mean 24-h, daytime, and night-time indicators have classically been the most used for both the relationship between ambulatory BP and cardiovascular prognosis, as well as for assessing the antihypertensive effect of drugs. Daytime BP, or BP during the period of activity, was one of the first parameters studied, since it is considered the closest to in-office BP. The current guidelines for diagnosing and treating HTN from the National Institute for Health and Care Excellence (NICE) of the British Government advises practicing ABPM during the day to confirm the HTN diagnosis when the clinic figures are high [8]. Daytime BP has also been the first used to assess the “white-coat” effect. In the first analyses of the Spanish ABPM Registry, up to 30% of patients without treatment [9, 10] and nearly 35% of those treated with clinic BP figures greater than or equal to 140/90 mmHg [11] presented normal daytime BP figures (below 135/85 mmHg).

One study has used daytime BP to guide antihypertensive treatment adjustment and monitoring in comparison with clinic BP [12]. Thus, patients with a clinic diastolic BP greater than or equal to 95 mmHg were randomized to using daytime BP or clinic BP to guide treatment adjustments. Upon ending the study, the daytime BP-guided monitoring was associated with a lower use of antihypertensive drugs; with no differences observed in the organ damage measured using the degree of left ventricular hypertrophy.

The mean BP of all measurements made over 24 h of monitoring is considered to be the one that provides better information. A larger number of measurements are included in its calculation. As such, it is less affected by sporadic situations that may arise during the day or night or by sporadic errors. It takes into account daytime activity, work, and the changes in pressure caused by those activities, as well as rest and the quality of it, and the night-time pressure dip. Its relationship with cardiovascular prognosis, as well as the presence and severity of organ damage, are clearly better than clinic pressure. From a therapeutic point of view, several meta-analyses have evaluated the correlation between the decrease in clinic BP or 24-h BP induced by antihypertensive treatment. Thus, in one of them that included 44 studies with more than 5000 patients, the mean decreases in clinic BP were 19/10 mmHg, while those corresponding to the 24-h figures were 13/8 mmHg. The percentage of 24-h BP reductions compared to clinic BP was 65% and 81%, respectively, for systolic and diastolic BP [13].

Another conclusion reached in that meta-analysis was that the definitions of responders and patients achieving BP control were not able to be extrapolated to the values obtained in 24-h ABPM. Thus, the final in-office BP values (143/90 mmHg) were only slightly better than the normal values (<140/90 mmHg), suggesting that a high percentage of patients managed to achieve BP control. Conversely, the final 24-h BP values (139/86 mmHg) were clearly above the limits of normal (<130/80 mmHg), suggesting that achieving 24-h control was far below the clinic control achievement. These results were later confirmed in another meta-analysis that included studies with clinic measurement, home measurement, and ABPM. The BP reduction was greater in the clinic than at home, and higher at home than in the 24-h values [14].

**Fig. 1:**
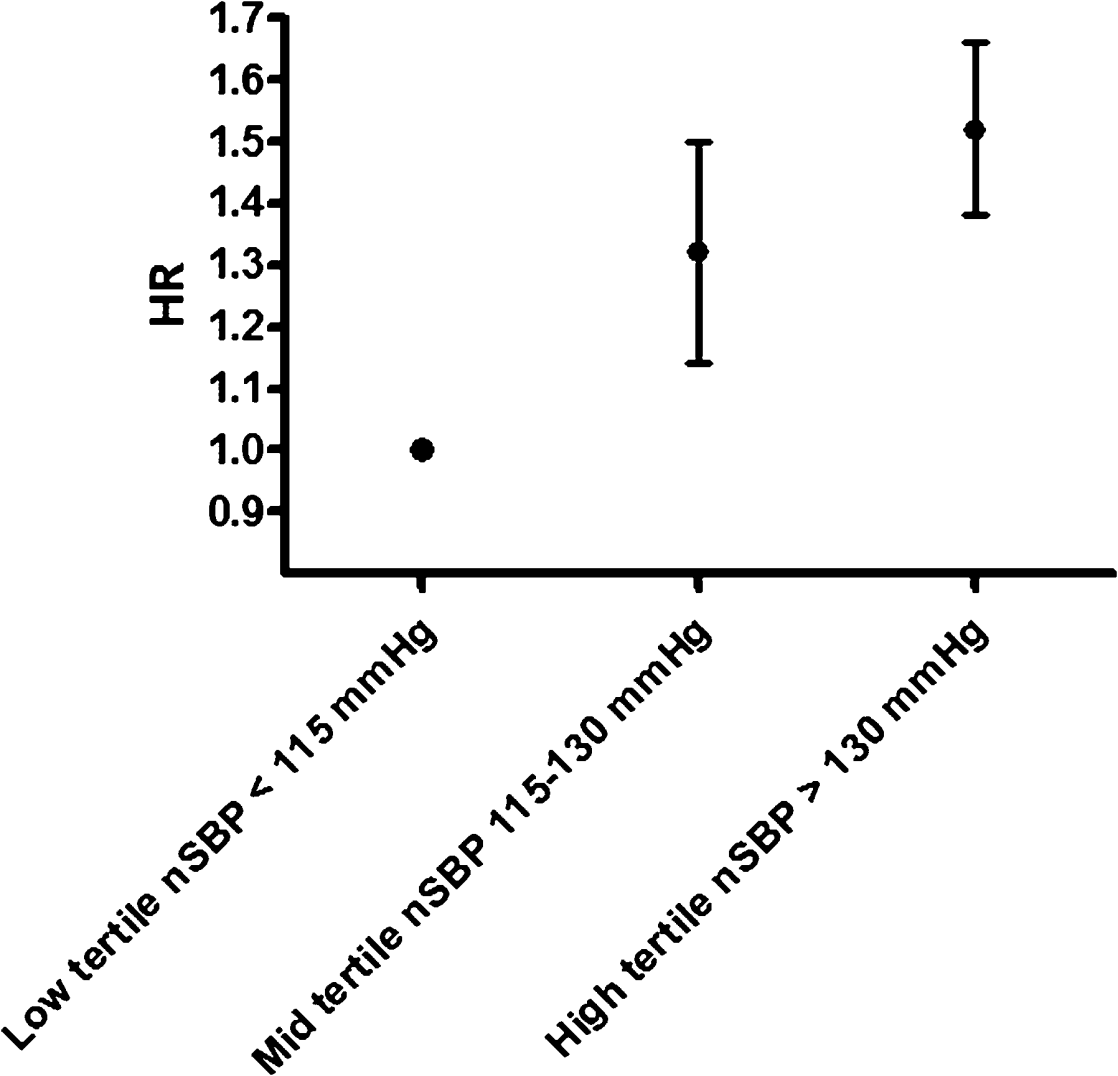
Risk of cardiovascular events (non-fatal myocardial infarctions, non-fatal strokes, or cardiovascular deaths) in high-risk patients based on the tertile distribution of nSBP. The risk increases 32% and 50%, respectively, for the *middle* and *high tertiles*. Data extracted from [4]. *nSBP* night-time systolic blood pressure, *HR* Hazard ratio

Night-time BP measurements have been progressively acquiring more importance. Of all the indicators obtained during ABPM, it is the one that is best correlated with the prognosis [1–4]. Its main advantage is that it can be considered the baseline BP (the one that is specified for tissue perfusion in a state of rest). In addition, the fact that it is generally measured at rest gives it higher reproducibility and less variability, which makes it easier to correlate it with organ damage and prognosis. The data from the Spanish ABPM Registry [4], as well as the various prospective cohort databases [15], indicate that of all the BP indicators (clinic, daytime, night-time, and 24-h) it is the one that is best independently correlated with the prognosis (Fig. 1). The main disadvantages are that it requires an exact definition of the rest period, it may be affected by the presence of a daytime rest (nap) [16], and it is equally affected by the quality of sleep [17], especially in patients who repeatedly wake up during the night or who have sleep apnea.

## Patient Phenotypes

Jointly using clinic measurement and ABPM has resulted in the creation of new patient categories that have expanded the hypertensive/normotensive dichotomy. Thus, the consistency between normal figures in clinic BP and those obtained through ABPM is called normotension, whereas the consistency between high figures in the clinic and ABPM constitute sustained HTN. The two new types represent the presence of discrepancies between both forms of measurement. Thus, high figures in the office and normal ones in ABPM constitute the phenotype known as white-coat HTN or isolated clinic HTN. This category, in principle, restricted to the diagnosis of patients without treatment, is also used in patients on treatment in whom ABPM figures are controlled, but not those in the clinic. At the opposite extreme are individuals with normal clinic BP figures but with high ABPM figures. This situation is known as masked HTN in untreated individuals or as masked uncontrolled hypertension (MUCH) in those who are on treatment.

Though there is a general consensus that the presence of masked HTN or MUCH confers a risk that may be comparable to that of sustained HTN [18, 19] (possibly from a population perspective the risk is even greater, given that the individuals who do not know they belong to this group are mostly not being treated and outside of health care oversight), there are still serious doubts about whether white-coat HTN carries an increased risk and whether it requires treatment [20, 21]. In general, longitudinal studies have observed that individuals with white-coat HTN present a risk of cardiovascular events similar to normotensive individuals, although in some cases, an increase in cerebrovascular accidents has been detected. Similarly, its association with organ damage has been described as similar to that of normotensive individuals in some studies, or with a higher prevalence of cardiac or renal damage in others. In many cases, it is hard to reach a conclusion since, even with the white-coat HTN diagnosis, these individuals have higher ABPM figures than normotensive individuals and, in addition, a very high percentage go on to develop sustained HTN in its progression [22].

**Fig. 2:**
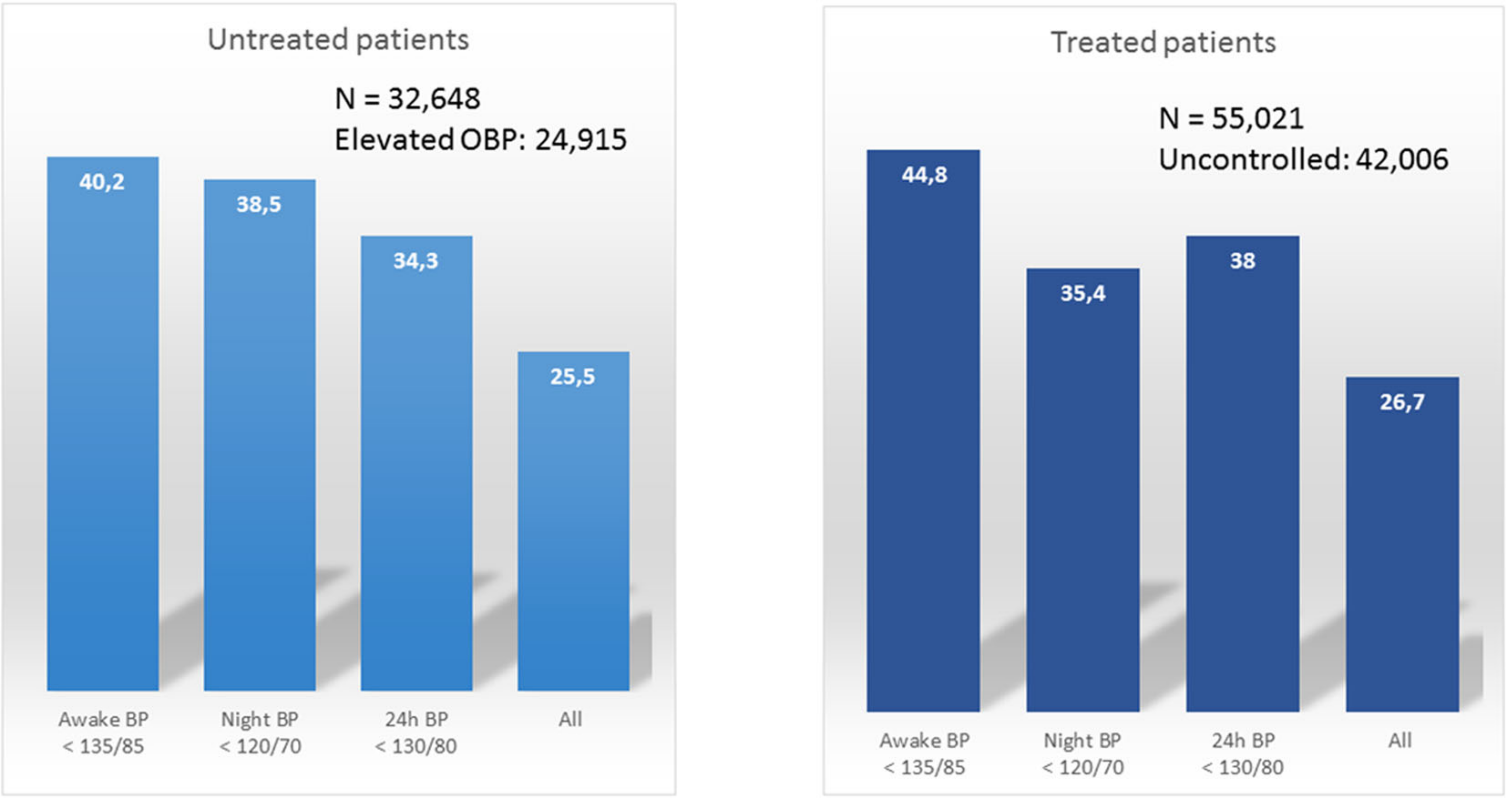
Prevalence of white-coat hypertension in patients in the Spanish Ambulatory BP Monitoring Registry with or without treatment and with clinic BP values greater than or equal to 140/90 mmHg. The prevalence depends on which parameter is used (daytime, night-time, or 24-h BP, or the normalcy all of them). *BP* Blood pressure, *OBP* Office blood pressure

**Fig. 3:**
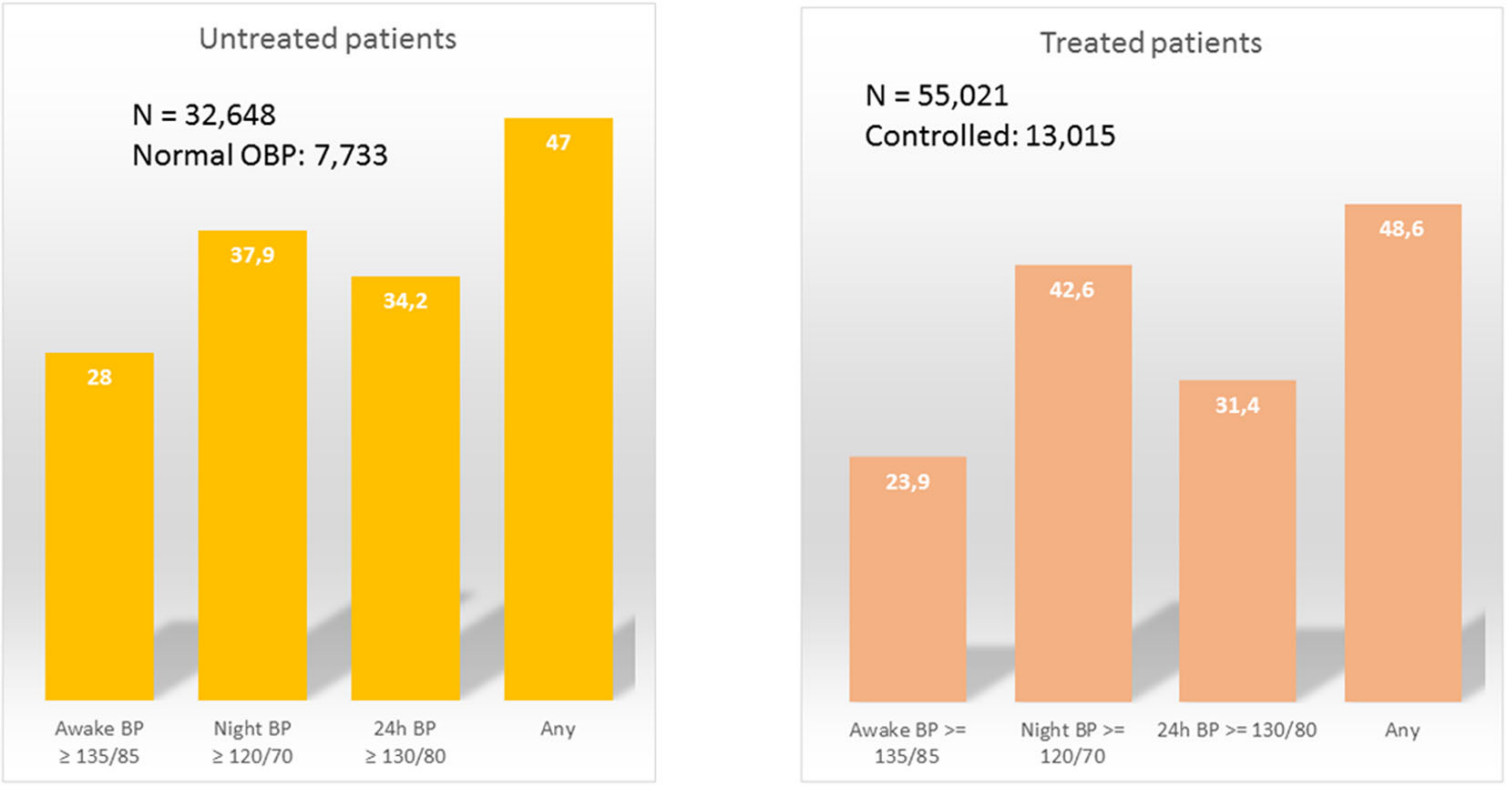
Prevalence of masked hypertension in patients in the Spanish Ambulatory BP Monitoring Registry with (*right*) or without (*left*) antihypertensive treatment and normal clinic BP (<140/90 mmHg). The prevalence depends on which parameter is used (daytime, night-time, or 24-h BP, or any of them). *BP* Blood pressure, *OBP* Office blood pressure

The prevalence of these divergent phenotypes depends in large part on the parameter used to define them and the study population. In patients with antihypertensive treatment and high clinic BP figures, white-coat HTN varies between 27%, if normality is required in all periods (daytime, night-time, and 24-h), and 45% if only daytime normality is considered [11] (Fig. 2). For its part, the prevalence of MUCH in patients with normal clinic BP figures also varies between 24%, if only daytime figures are considered, and 49% if it is defined based on elevation in any of the indicators (daytime, night-time, or 24-h; Fig. 3) [23].

The clinical features that are associated with these divergent phenotypes are, in the case of white-coat HTN, old age, the female sex, the absence of other risk factors such as smoking or diabetes, and cardiac or renal organ damage [9, 24]; whereas youth, the male sex, smoking, diabetes, and the presence of organ damage are correlated with a higher probability of presenting masked HTN [23, 25]. Obesity deserves particular mention, as it seems to be correlated, albeit weakly, with both white-coat HTN and masked HTN [9, 11, 23]. The interpretation is that obesity may be an element that decreases the accuracy of clinic measurement and, as such, has an impact not just on phenotype, but also on discrepancies between measurements. However, it should be recognized that these clinical associations, although significant, have little predictive ability in individuals and in no case should substitute ABPM in diagnosing a specific patient. Other features or the physician’s intuition should also not have any impact. Thus, the sensitivity and specificity of the clinical suspicion related by the physician for diagnosing white-coat HTN presented very low values in relation to the definitive diagnosis made by using ABPM [9].

At this time, there is no absolute consensus on the need for antihypertensive treatment in patients with divergent phenotypes. All the observational studies demonstrate that masked HTN or MUCH result in a high risk of developing future cardiovascular events. As such, it seems logical to think that those patients are suitable for starting antihypertensive therapy. Nevertheless, no study has demonstrated that this treatment will improve the prognosis in those patients; therefore, the therapeutic decision is completely empirical. Even more uncertainties exist about the follow-up for these patients, including target figures or regularity of repeating ABPM [26].

As for subjects with white-coat HTN, as previously mentioned, there are discrepancies between the likelihood of having a major cardiovascular event or not. Moreover, treatment in those patients, even reducing the BP figures, seems to have little impact on the BP figures obtained by ABPM. The current guidelines recommend antihypertensive treatment in high-risk patients with known cardiovascular disease or with hypertensive organ damage. In the rest, a close follow-up and early detection of the appearance of sustained HTN seems to be the best option [27].

## Dipping Patterns

The decrease in BP caused by rest and sleep, usually at night, has a favorable impact on reducing the pressure burden related to the organ damage. Almost 40 years ago, it was described that some patients in whom this night-time dip was less pronounced (the threshold has been established at 10% versus the daytime values) had a worse risk profile and a higher probability of developing cardiovascular events and death [28]. In general, four dipping patterns have been described based on this night-time decrease. The most common pattern in the healthy population is known as the “dipper” pattern and it represents between a 10% and 20% decrease from daytime values. The extreme “dipper” pattern exceeds this 20% and, even though it has been described as associated with a risk of cerebrovascular accident in the Asian population, a clearly deleterious effect has not been demonstrated on the prognosis in Westerners. Conversely, a decrease below 10%, known as a “non-dipper” pattern (recently the term “reduced dipper” has been proposed), or an increase in BP during rest, known as a “riser” pattern, have both been associated with a worse prognosis and related with organ damage [28, 29].

**Fig. 4:**
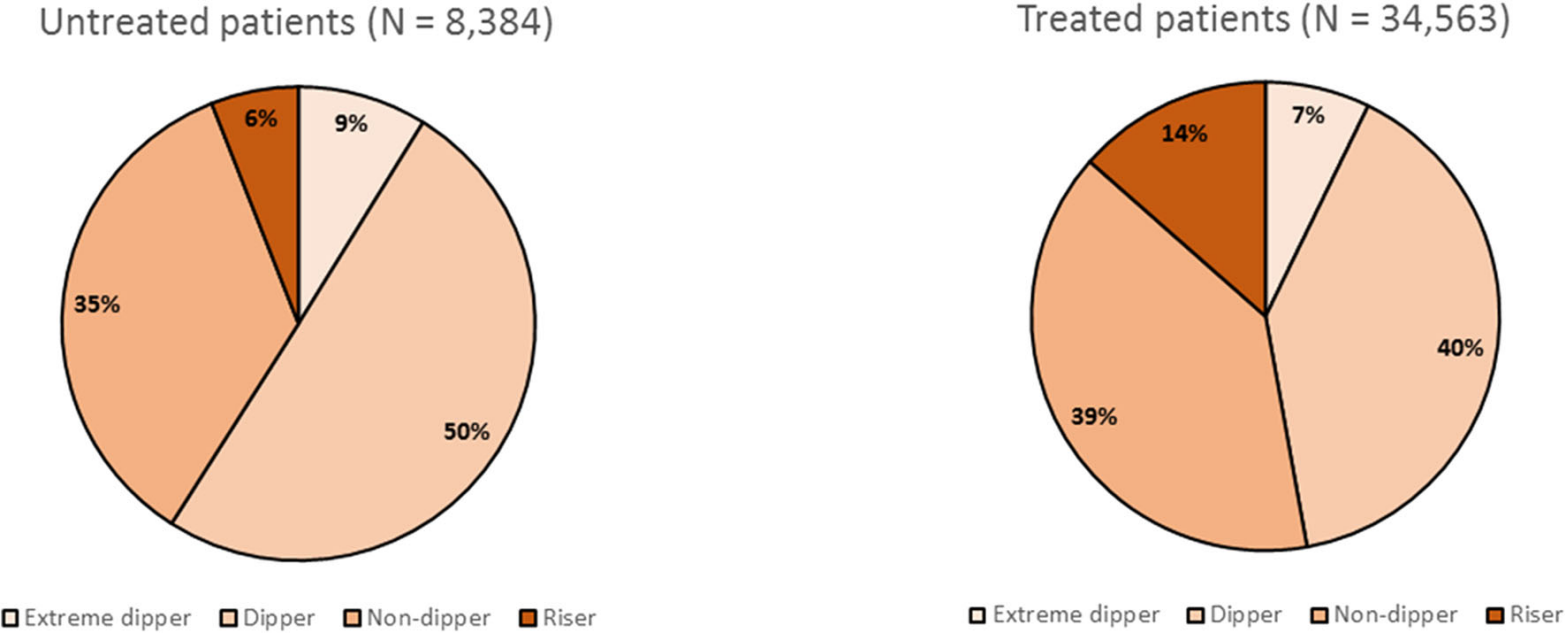
Distribution of dipping patterns in patients without hypertensive treatment (*left*) or with hypertensive treatment (*right*) included in the Spanish Ambulatory Blood Pressure Monitoring Registry. The presence of an inadequate night-time dip (riser or non-dipper pattern) is around 50% in patients without treatment and exceeds this figure in those treated

The data from the Spanish ABPM Registry have allowed us to determine that the prevalence of these “deleterious” patterns is very high, approaching 50% of untreated patients and exceeding this figure in those on treatment (Fig. 4). Old age, the female sex, obesity, diabetes, and a history of previous cardiovascular disease are associated with an inadequate decrease in both treated and untreated patients. In treated patients, increasing the number of drugs also results in a higher probability of presenting a non-dipper or riser pattern [30, 31].

The main problem in assessing the dipping pattern is its association with night-time BP levels. Both elevated night-time BP as well as inadequate nocturnal dip have been related with a worse prognosis. Nevertheless, the fact remains that both situations are intimately related, which makes it difficult to separate their independent effects. A separate assessment of both phenomena has enabled these uncertainties to be cleared up. Thus, there have been patients with inadequate night-time BP dip who despite everything present normal night-time BP figures, and by contrast, there have been patients with night-time HTN but with a night-time BP dip above 10%. In this analysis, the non-dipper pattern in the absence of night-time HTN was associated with the female sex, impaired kidney function, and a history of cardiovascular events, whereas night-time HTN in the presence of a normal pattern was associated with the male sex, diabetes, and asymptomatic organ damage (microalbuminuria and left ventricular hypertrophy). Obviously the worse risk profile was observed in patients who presented both night-time HTN and a non-dipper pattern [32].

## Morning HTN and Morning Surge

After the sleep-induced night-time BP dip, the morning surge that accompanies waking is a physiological phenomenon. However, some studies have observed that an exaggerated morning BP surge is associated with a higher rate of cardiovascular events [33]. The hormone changes that affect cortisol and catecholamines, the increase in heart rate, and the higher platelet aggregability that happen in these morning hours are a rationale to explain this phenomenon [34].

Another parameter of interest intimately related to the morning surge is known as morning HTN, which consists of high BP figures obtained just after waking. A recent study in patients being treated for HTN demonstrated that the BP figures obtained in the first hour of the morning through self-measurement had a greater prognostic impact than clinic BP figures. Values above 145 mmHg were associated with a higher rate of cardiovascular events [35].

The main problems that arise when evaluating the phenomena of the morning surge and morning HTN are, on one hand, that most studies have been carried out in a Japanese population. Data in a Mediterranean population suggest that the morning surge in this population is less pronounced than in the Japanese population (De la Sierra; personal communication); thus its prognostic impact is presumably less. On the other hand, morning surge and morning HTN are influenced not only by physiological or pathophysiological circumstances, but also by type of treatment, its posology, and the duration of action of the drugs. Thus, if we take into account that most drugs are administered in the morning, this morning surge coincides with the end of the period of the therapeutic window, and therefore only those drugs with a longer half-life will significantly reduce these parameters.

Although there are not many comparative studies, not all antihypertensive drugs ensure 24 h of coverage. Olmesartan and telmisartan among the angiotensin receptor blockers, lisinopril among the angiotensin-converting enzyme inhibitors, amlodipine among the calcium blockers, and chlorthalidone among the diuretics are probably the drugs with the longest duration of antihypertensive effect in their respective classes. Some others with shorter coverage times may see that time extended with pharmaceutical modifications that slow down their absorption.

## BP Variability

BP is a dynamic parameter that fluctuates based on several circumstances, some intrinsic and other extrinsic [36]. Long-term BP variability may be determined through successive visits. This variability has a prognostic impact, especially in predicting cerebrovascular accidents [37]. ABPM enables short-term fluctuations to be assessed. Some of these, caused by the activity/rest rhythm, have already been mentioned and are part of the night-time dip patterns. However, pressure fluctuations within one of these periods (daytime and especially at night) also have a prognostic impact. They can be evaluated by calculating the standard deviation in one of the periods separately and by using indices that take into account these deviations, and project the calculation over the entire 24-h period [36].

In recent years, there has been a growing interest in assessing the impact of antihypertensive treatment on this short-term variability determined by ABPM. Initial studies have used modifications in the posology of the drugs (administering part or all of the treatment at night) and assessed their impact on the dipping pattern. Thus, it has been demonstrated that this night-time administration promotes a larger night-time dip and, as such, a proportion of non-dipper or riser patients becomes dippers. In one of these studies, this phenomenon was associated with a better cardiovascular prognosis [38].

## Therapeutic Indices Obtained Using ABPM

In addition to all the above-described parameters used to assess the effect of antihypertensive therapy, some mathematical indices have been developed that combined potency, duration of action, and homogeneity of effect. This allows for a better assessment of the effects of antihypertensive treatment.

The first of these indices is the trough-to-peak (*T*/*P*) ratio. Interest in this ratio appeared nearly two decades ago and consists of calculating the ratio between the decrease in BP obtained in the hours just before the end of the therapeutic window (with drugs administered once a day between 22 and 24 h after administration) and the maximum effect calculated after several hours (between 4 and 6 h after administration). In theory, the closer it is to unity, the greater the homogeneity of effect, suggesting that the residual effect of the drug is close to its maximum effect. However, the *T*/*P* ratio has two significant problems. The first is a result of poor reproducibility, caused by the need to extract short periods of monitoring from a 24-h ABPM that may be influenced by external factors. Thus, the peak period or maximum effect may coincide with a postprandial rest (nap), which will magnify it, or with a period of higher physical or mental activity, which will minimize it. For its part, calculating the trough effect may coincide with the last hours of sleep or with waking, both circumstances that may change it. In addition, the *T*/*P* ratio does not take into account the magnitude of the antihypertensive effect, so that minimal decreases in BP in the peak will be associated with high *T*/*P* indices (placebos usually have a *T*/*P* index around 1) [39].

The second index that measures homogeneity of effect is called the smoothness index (SI) [40]. It is calculated based on the hourly reductions in BP, corrected by the standard deviation of those reductions. Thus, the greater the magnitude of the hourly reduction and the smaller the differences between those reductions (less variability), the higher the resulting number will be. The higher the SI value is, the higher the drug potency and the greater the homogeneity of effect. Some studies have described an ability of the SI to predict changes in organ-damage parameters (left ventricular mass and carotid intima-media thickness) caused by the treatment [41].

One last index proposed very recently is the treatment-on-variability index (TOVI), calculated using the ratio between the 24-h decrease in BP and the change in the weighted standard deviation (hourly standard deviation calculated separately during the daytime and night-time periods and later weighted based on the duration of each of those periods) [42]. A recent study using clinical trial databases with several monotherapies and one combination therapy demonstrated a greater effect of the combination therapy and the amlodipine monotherapy based on that index, in comparison with two angiotensin receptor blockers and one angiotensin-converting enzyme inhibitor [42].

## Resistant HTN

Between 10% and 15% of patients with HTN do not manage to normalize their BP values despite treatment with 3 or more antihypertensive drugs. They fall under the category of resistant HTN [24, 43]. Most of them are referred to a specialist clinic, and despite extensive diagnostic work and a search for secondary causes that explain their high BP, the reason why the BP values cannot be normalized has not been explained. These patients have more organ damage [44] and a worse prognosis [45] than other patients with HTN.

In a broad study stemming from patients in the Spanish ABPM Registry, one-third of 8295 patients who could be categorized as resistant hypertensive had normal 24-h BP figures, where resistance was due to a white-coat effect. Compared with truly resistant hypertensive patients, the organ damage in these patients was less and the cardiovascular prognosis better [24].

The need to demonstrate that the BP values obtained using ABPM are undoubtedly high in resistant hypertensive patients was recently supported by the appearance of more invasive therapies, such as renal sympathetic denervation or baroreflex stimulation as a treatment for patients with resistant HTN [46]. The first studies on renal denervation have demonstrated a significant decrease in BP that was not confirmed in the recent SIMPLICITY HTN-3 study (ClinicalTrials.gov number, NCT01418261), where patients with resistant HTN required ABPM confirmation [47].

## Conclusion

ABPM should be considered the standard measurement of BP, a starting point for assessing and treating patients with HTN, as some recent guidelines recommend [5, 6, 8, 48]. A large body of evidence demonstrating a close epidemiological relationship between the indicators obtained using ABPM, organ damage, and cardiovascular prognosis has not been followed by an equally significant translation of the impact of ABPM as a guide to antihypertensive therapy. It has only been in recent years where, in part due to scientific interest and in part due to requirements from regulatory agencies, the need for assessing the effects of the primary treatments on 24-h BP has been emphasized. The consistency of the mean estimators (24-h, daytime, and night-time BP figures), the new phenotypes of white-coat HTN and masked HTN, the importance of the dipping status and BP variability, and the appearance of specific indices for treatment assessment have made it so that ABPM should today be considered an essential element for guiding antihypertensive treatment, thereby enabling a more personalized medicine adapted to the patient. The main barriers for a more widespread use of ABPM are related to several factors including costs and reimbursement, acceptability and complexity in the interpretation of some estimators. However, they can be easily solved. More validated devices are available in the market with reduced prices, and the acceptance of patients and health workers in considering results as a better guidance for diagnosis and treatment has considerably increased. Moreover, different software and website platforms have developed to provide rapid and easy reports containing the most important estimators of clinical validity. Some experiences in several countries, including Australia, Ireland, Italy, and Spain have demonstrated that ABPM could be implemented in almost all clinical settings, from primary care to reference units, and even in community pharmacies. This will improve HTN management in the very near future.

## Electronic supplementary material

Below is the link to the electronic supplementary material.

Supplementary material 1 (PDF 200 kb)
