# Supplementary material for: Advantages of Ambulatory Blood Pressure Monitoring in Assessing the Efficacy of Antihypertensive Therapy
Source: Cardiol Ther. 2015 Jun 16;4(Suppl 1):5–17. doi: 10.1007/s40119-015-0043-1 (PMC6430151; doi:10.1007/s40119-015-0043-1)
Supplement: Supplementary file 1 — Supplementary material 1 (PDF 200 kb) [file 40119_2015_43_MOESM1_ESM.pdf]

Cardiology and Therapy
OPEN ACCESS
PEER REVIEWED SUMMARY SLIDE

- Ambulatory blood pressure monitoring (ABPM) is superior to office blood pressure (BP) in predicting organ damage and cardiovascular prognosis.
- ABPM also constitutes an important tool in evaluating the effect of antihypertensive therapies.
- Twenty-four-hour, daytime, and night-time BP, the presence of new phenotypes, such as “white-coat” and “masked” hypertension, nocturnal BP dip and BP variability measures, are all important prognostic variables, which also contribute to a better guidance of antihypertensive treatment.
- There are specific ABPM-derived therapeutic indexes, such as the “through-to-peak ratio”, the “smoothness index”, and the “treatment-on-variability” index which give important information with respect to the effect of antihypertensive treatment on both effectiveness, as well as the homogeneity of the therapeutic effect.

This summary slide represents the opinions of the authors. This supplement has been sponsored by Menarini. For a full list of acknowledgments and conflicts of interest for all authors of this article, please see the full text online. Copyright © The Author(s) 2015. Creative Commons Attribution Noncommercial License (CC BY-NC).
